# Hepatitis C reinfection in former and active injecting drug users in Belgium

**DOI:** 10.1186/s12954-021-00552-x

**Published:** 2021-10-12

**Authors:** Dana Busschots, Rob Bielen, Özgür M. Koc, Leen Heyens, Rita Verrando, Chantal de Galocsy, Christophe Van Steenkiste, Frederik Nevens, Håvard Midgard, Olav Dalgard, Geert Robaeys

**Affiliations:** 1 Faculty of Medicine and Life Sciences, Hasselt University, Martelarenlaan 42, Diepenbeek, 3500 Hasselt, Belgium; 2 Department of Gastroenterology and Hepatology, Ziekenhuis Oost-Limburg, Genk, Belgium; 3 School of NUTRIM, Maastricht University Medical Centre, Maastricht, The Netherlands; 4 zorGGroep Zin, Hasselt, Belgium; 5 Private practice, 1190 Brussels, Belgium; 6 Department of Gastroenterology and Hepatology, AZ Middelares, Gent, Belgium; 7 Department of Gastroenterology and Hepatology, University Hospitals KU, Leuven, Belgium; 8 Department of Infectious Diseases, Akershus University Hospital, Lørenskog, Norway

**Keywords:** Reinfection, Hepatitis C virus, People who inject drugs, Direct-acting antivirals

## Abstract

**Background:**

There is currently no systematic screening for hepatitis C (HCV) reinfection in people who inject drugs (PWID) after treatment in Belgium. However, in a recent meta-analysis, the overall HCV reinfection rate was 5.9/100 person-years (PY) among PWID. Accordingly, this study was undertaken to investigate the reinfection rate in former and active PWID who achieved the end of treatment response after direct-acting antiviral (DAA) treatment in Belgium.

**Methods:**

This observational cross-sectional study recruited individuals with a history of injecting drug use who had achieved the end of treatment response to any DAA treatment between 2015 and 2020. Participants were offered a post-treatment HCV RNA test.

**Results:**

Eighty-five potential participants were eligible to participate and contacted, of whom 60 participants were enrolled in the study with a median age of 51.0 (IQR 44.3–56.0) years; it was reported that 23.3% continued to inject drugs intravenously after DAA treatment. Liver cirrhosis was present in 12.9%. The majority had genotype 1a (51.7%) or genotype 3 (15.0%) infection. We detected no reinfections in this study population. The total time patients were followed up for reinfection in the study was 78.5 PY (median 1.0 years IQR 0.4–2.0).

**Conclusion:**

Reinfection after successful treatment with DAA initially appears to be very low in Belgian PWID. Therefore, efforts should be made to screen individuals with persistent risk behaviors for reinfection systematically. In addition, a national HCV registry should be established to accurately define the burden of HCV infection and reinfection in Belgium and support the elimination of viral hepatitis C in Europe.

*Trial registration* clinicaltrials.gov NCT04251572, Registered 5 Feb 2020–Retrospectively registered, https://clinicaltrials.gov/ct2/show/NCT04251572.

## Background

The mortality rate resulting from infection with the hepatitis C virus (HCV) is significant due to complications (e.g., liver cirrhosis and hepatocellular carcinoma) [1]. Approximately 1.0% or 71.1 million (62.5–79.4) people worldwide were chronically infected with HCV in 2015 [2]. The estimated prevalence of HCV antibodies (Ab) and HCV-RNA in Belgium is 0.22% and 0.12%, respectively [3]. These relatively low results are consistent with the Ab prevalence in other Western European countries, particularly in neighboring countries such as France (0.8%), the Netherlands (0.22%), and Germany (0.4%) [4–7]. However, subgroups with higher HCV prevalence exist, and people who inject drugs (PWID) are the most important [8]. A recent study estimated the HCV Ab prevalence in Belgian PWID at 41.1% similar to data found in France (43.4%), the Netherlands (39.3%), and the UK (49.1%) [4, 9, 10].

The introduction of highly effective direct-acting antiviral (DAA) therapy has changed the HCV treatment paradigm. These changes led to progress toward achieving the World Health Organization's stated goal of reducing HCV viral infections by 90% (specifically 80% reduction in new HCV cases) and mortality by 65% by 2030 [11, 12]. However, the high list price of DAAs has led many governments to impose certain reimbursement conditions, such as fibrosis stage, drug or alcohol use, prescriber type, and HIV coinfection [13]. DAA regimens have been available in Belgium since 2015 and fully reimbursed for all stage fibrosis since 2019 [14, 15]. However, there are still some restrictions regarding reimbursement of DAA medication. Currently, treatment for HCV is only reimbursed if the patient has a chronic infection (> 6 months). However, individuals are infectious even during those first six months and thus can cause ongoing transmission, especially in high-risk groups such as active PWID. Also, DAA treatment can only be prescribed by a hepatologist and is only available in a hospital pharmacy [15].

DAA treatment is also effective in individuals who have received opioid agonist treatment (OAT) and active PWID. Nevertheless, providing HCV care in this population can be challenging because of the loss of follow-up and reinfection after successful treatment in cases of persistent risk behavior [16].

Reinfection is a major concern because it can potentially endanger both the individual and population benefits of HCV treatment. Timely detection of reinfections will also help stop persistent transmission of the virus. In a recent meta-analysis including studies from the DAA era, the overall HCV reinfection rate was 3.9/100 person-years (PY) among PWID [17]. Reinfection data are scarce in Belgium. In 2017, the reinfection rate in one Belgian addiction care center was 2.6/100 PY over a follow-up time of 39 PY [18]. Although it is acknowledged that regular HCV RNA testing after treatment in PWID is an indispensable part of any elimination strategy, the feasibility of monitoring for reinfection and retreatment has been little explored [16]. Also, there is no national HCV registry in Belgium, making it difficult to follow patients after treatment. This study was undertaken to investigate the reinfection rate of HCV in PWID after successful treatment with DAAs in Belgium. In addition, we wanted to expose the gaps in HCV care in the Belgian PWID population. From these perspectives, recommendations can be made for HCV care post-treatment in other regions.

## Methods

### Study design and participants

This observational cross-sectional study recruited individuals aged 18 years or older with a history of injecting drug use (IDU) who had achieved the end of treatment response (defined as non-detectable HCV RNA at the end of treatment) to any interferon-free DAA treatment between 2015 and 2020. The study was conducted in three centers. There were two centers in Flanders, one out of hospital drug addiction center and the digestive department of a hospital. The third center, a hepatologist private practice (out of hospital), was in Brussels.

The local researchers of the cooperating centers contacted the potential study participants and invited them to participate in the study between August 2019 and December 2020. A blood draw was done during the study visit for HCV RNA determination. Potential HCV risk factors were assessed through a face-to-face questionnaire on paper in a private and secure setting. The questionnaire was available in Dutch, French, and English and covered a total of 29 questions. Data from the questionnaire included birth gender, year of birth, source of income, level of education, housing in the past six months, history of imprisonment, tattoos or piercings placed in a potentially non-sterile environment, number of unsafe sexual partners, frequency of alcohol abuse (> 14 units per week for women or > 21 units per week for men [19]), age of first drug use, kind of drugs injected and when (ever, after DAA treatment), frequency of IDU after DAA treatment, having shared paraphernalia, OAT, and needle syringe program (NSP). If available in the medical record, the results of the FibroScan® (cutoffs for HCV: F0–F1 =  < 7.2 kPa, F2 = 7.2–9.5 kPa, F3 = 9.5–12.5 kPa, F4 =  > 12.5 kPa) were noted [20].

The ethical committee of Ziekenhuis Oost-Limburg approved the study protocol on August 23, 2018 (18/0012U). The study was conducted following the provisions of the Declaration of Helsinki and its amendments. Good clinical practice guidelines were followed throughout the study, and all participants provided written informed consent [21].

### Outcome

Reinfection was defined as HCV RNA recurrence following virologic response at the end of treatment [16]. To optimize the follow-up of reinfection in PWID, the gaps in current care were exposed.

### Statistical analysis

The primary outcome was the prevalence (%) of HCV reinfection. Patient demographics were summarized using mean ± standard deviation for continuous characteristics and by proportions for categorical characteristics. All analyses were performed using IBM SPSS Statistics 25.

## Results

**Fig. 1:**
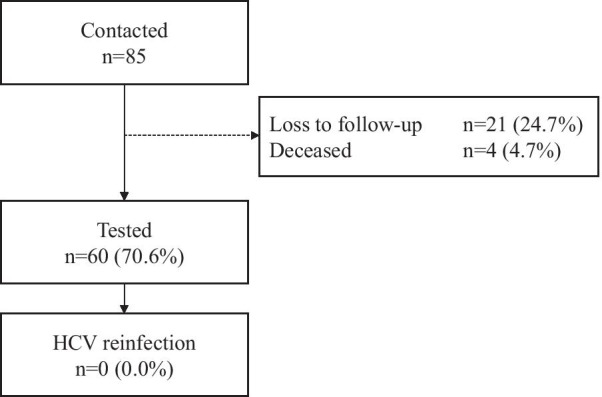
Flowchart of the study

Between August 2019 and December 2020, 85 potential participants were eligible to participate and contacted by the local investigators. Twenty-one individuals could not be contacted and were lost to follow-up (reason unknown), and four individuals had passed away. Sixty (70.6%) individuals were enrolled in the study (Fig. 1).

At the time of inclusion, the median age was 51.0 (44.3–56.0) years, and 81.7% were male. The sociodemographic characteristics of the study population are shown in Table 1.

**Table 1:** Sociodemographic characteristics of the participants

Characteristics	*N* (%)
Gender	
Male	49 (81.7)
Female	11 (18.3)
Median age (IQR)	51.0 (44.3–56.0)
Age groups	
< 30	2 (3.3)
30–39	5 (8.3)
40–49	19 (31.7)
50–59	31 (51.7)
≥ 60	3 (5.0)
Birth country	
Belgium	51 (85.0)
Other	9 (15.0)
Income	
Employed	12 (20.0)
Temporary benefit (unemployed)	15 (25.0)
Temporary benefit (disability)	31 (51.7)
Retirement	1 (1.7)
No income	1 (1.7)
Education	
Completed primary	13 (21.7)
Partly high school	25 (40.0)
High school	18 (30.0)
University/college	5 (8.3)
Housing	
Owned/rented	48 (80.0)
Residential	3 (5.0)
Family/friends	8 (13.3)
Homeless	1 (1.7)
Household member with HCV	
Yes	13 (21.7)
No	47 (78.3)
History of imprisonment	
Yes	41 (68.3)
No	19 (30.6)
Potentially non-sterile tattoo/piercing	
Yes	28 (46.7)
No	32 (53.3)
Unsafe sexual contacts	
Yes	59 (98.3)
No	1 (1.7)
Number of unsafe sexual contacts	
1–5	12 (20.3)
6–10	10 (16.9)
> 10	15 (25.4)
Missing	22 (37.3)
Alcohol abuse	
Never	9 (15.0)
In the past	15 (25.0)
< Monthly	14 (23.3)
< Weekly	6 (10.0)
> Weekly	6 (10.0)
Daily	10 (16.7)

‘Unsafe’ refers to sexual contact without the use of a condom

IQR, interquartile range; HCV, hepatitis C virus

All participants had a history of IDU, 71.7% (*n* = 43) received OAT, 21.7% (*n* = 13) injected drugs during treatment, and it was reported by 23.3% (*n* = 14) after DAA treatment (Table 2). Among those that injected after treatment, 50.0% (*n* = 7) reported at least weekly injecting. The most frequently injected drug was heroin (71.4%).

**Table 2:** Characteristics concerning injecting drug use (*n* = 60)

Characteristics	*N* (%)
Median age first drug use (IQR)	18.0 (16.0–21.0)
OAT	43 (71.7)
NSP	45 (75.0)
Ever heroin	55 (91.7)
Ever amphetamines	7 (11.7)
Ever cocaine	28 (46.7)
Ever shared a needle	45 (75.0)
IDU during DAA	13 (21.7)
IDU after DAA	14 (23.3)
Heroin	10 (71.4)
Amphetamine	3 (21.4)
Cocaine	4 (28.6)
How often IDU after DAA (*n* = 14)	
Less than weekly	7 (50.0)
More than weekly, not daily	7 (50.0)
Daily	0
Shared a needle after DAA (*n* = 14)	7 (50.0)
Frequency (*n* = 7)	
Once/twice	4 (57.1)
Sometimes	3 (42.9)

IQR, interquartile range; IDU, intravenous drug use; OAT, opioid agonist therapy; NSP, needle syringe program; DAA, direct-acting antivirals

Liver fibrosis scores were available in 96.7% (*n* = 58), and 12.9% (*n* = 8) had liver cirrhosis (F4). The majority had genotype 1a (51.7%, *n* = 31) or genotype 3 (15.0%, *n* = 9) infection (Table 3).

**Table 3:** Medical characteristics of the participants

Characteristics	*N* (%)
HIV coinfection	0
Fibrosis	
F0–F1	27 (43.5)
F2	12 (19.4)
F3	13 (21.0)
F4	8 (12.9)
Missing	2 (3.2)
Genotype	
1a	31 (51.7)
1b	7 (11.7)
3	9 (15.0)
4	5 (8.3)
Missing	8 (13.3)
Year end of treatment	
2015	1 (1.7)
2016	6 (10.0)
2017	7 (11.7)
2018	9 (15.0)
2019	30 (50.0)
2020	7 (11.7)

In this cohort, we detected no reinfections. The total time patients were followed up for reinfection in the study was 78.5 PY (median 1.0 years IQR 0.4–2.0).

## Discussion

This study evaluated HCV reinfection in 60 PWID in three centers in Belgium. Although about one-quarter of the population had still been actively using drugs, no reinfections were detected in this study group.

Reinfection in PWID after DAA treatment is generally relatively low. In two international, multicenter trials (SIMPLIFY and D3FEAT), participants were enrolled at 25 sites in Australia, Canada, France, New Zealand, Norway, Switzerland, the UK, and the USA. The researchers observed a reinfection prevalence of 4.5% and an overall reinfection rate of 3.1/100 PY [22]. In a recent study in Norway, the prevalence of HCV reinfection was 2.7% (8/297) and the incidence 2.60/100 PY (95% CI 1.12–5.11)[16]. Also, in Scotland, reinfection in people living with HIV (80% PWID) appears to be notably low, with an incidence of 0.2/100 PY [23]. In a recent meta-analysis including studies from the DAA era, the overall HCV reinfection rate was 5.9/100 PY (95% CI 4.1–8.5) among PWID [17]. However, we must keep in mind that the reinfection rate depends on the context. It may depend on how quickly DAA treatment is scaled up and the proportion of the high-risk population that receives treatment [24]. Large, high-quality multicenter studies are needed to get an accurate overview of this problem.

Our findings are also lower than data from previous Belgian studies. In one study, the prevalence was 2.8%, and the incidence was 2.6/100 PY. However, this study was monocentric and with a limited number of inclusions (36 participants, one reinfection) and a shorter follow-up time (39 PY)[25]. Nevertheless, there were two previously detected reinfections (personal communication; [17]). We thus arrive at a prevalence of 2.8% with two reinfections in 71 patients after treatment with a sustained virologic response in the province of Limburg, Belgium. However, we have insufficient data to calculate the exact incidence. The incidence in another (monocentric) study with five reinfections was 0.5/100 PY which is an exceptionally low incidence given that 60.0% had injected drugs after treatment [26]. However, similar to our findings, the mean age in this cohort was also 51 years which could partially explain the low reinfection rates. Studies have shown that younger individuals had higher reinfection rates, potentially due to higher rates of risk behavior (e.g., needle sharing) [16, 27]. In addition, the low reinfection prevalence in our study could also be explained by the fact that recent IDU was low (23.3%), and daily injection was even completely absent. This finding is not surprising since most of the participants are from rural areas (province of Limburg). In rural settings, the frequency of injecting use is often lower than in a (metropolitan) city [28]. In addition, in the meta-analysis of Hajarizadeh et al., recent IDU varied between 17.0 and 100%, and in the Norwegian study and the study of Cunningham et al., recent IDU was remarkably high, 70.1% and 73.0%, respectively [16, 17]. Furthermore, we suspect that we were unable to reach those at the highest risk (active injectors) because they were unable to contact them, and the follow-up period was relatively short.

The literature states that reinfection is slightly lower in users receiving opioid agonist therapy (OAT) with an incidence of 3.8/100 PY (95% CI 2.5–5.8) [17]. In a study at an OAT clinic in the Bronx, New York, a reinfection prevalence of 2.1% and a reinfection rate of 1.2/100 PY were measured in PWID who received OAT [29]. The absence of reinfection in our cohort may reflect the relatively short follow-up time and the effect of an integrated model of care with good access to OAT and NSP, combined with high reinfection awareness among study participants. OAT and NSP coverage in our cohort was high, respectively, 71.7% and 75.0%, and similar to other European countries [16, 17]. These findings align with Hajarizadeh et al. and Rossi et al., who showed a lower reinfection risk among people receiving OAT [17, 30].

Besides, there is no systematic screening or follow-up for reinfection in high-risk groups. As Midgard et al. showed in their study, reinfection surveillance and retreatment in a real-world PWID cohort is feasible [16]. Although this is a single-center study, it is questionable whether this is feasible on a national level. This brings us to one of the major shortcomings in terms of HCV care in Belgium, the lack of a national registry for HCV. A national registry could map the progression to elimination as all initiatives are currently at a local level. This could drastically reduce the lost-to-follow-up and provide the opportunity for concrete monitoring of patients. For proper follow-up of patients after treatment, not only a national registry but also an HCV case manager is essential. Follow-up by an HCV case manager is beneficial not only for follow-up of reinfection but also for liver cirrhosis. This is equally important, especially since we observe that liver cirrhosis is present in 12.9% in this group. The case manager can work closely with addiction care centers and hepatologists in a hospital. In Limburg, Belgium, the zorGGroep Zin has been working with an HCV case manager since 2015. This has dramatically increased the rate of screening, treatment, and follow-up after treatment [31]. Consequently, the lack of a specific person focusing solely on HCV care was probably the main reason for not participating in this study due to the high workload of the current staff.

This study had several limitations. First, we did not actively follow this group. As a result, spontaneous clearances could not be detected, and we, therefore, underestimated the actual burden. Moreover, it was a cross-sectional study. As a result, we were unable to calculate the PY of follow-up correctly. Although we had the end of treatment date, we could not be sure if the individual had been tested in the meantime, which led to the fact that we could only report the reinfection prevalence. Given the small number of participants, these data may not be generalizable to all PWID in Belgium. Furthermore, the inclusion rate is subject to the COVID measures in place in Belgium since March 13, 2020. As a result, regular care was drastically reduced, and scientific studies were temporarily put on hold. Another fact that has had a negative impact on our inclusion rate is the lack of a national register. This is not in line with HCV elimination, as we cannot provide accurate national data. We must also consider selection bias. Individuals could choose to participate in this study. Therefore, it is possible that active users with a high-risk profile did not participate. In addition, data were also not collected from individuals who declined to participate or from those who were lost to follow-up. This potential selection bias may also have contributed to the fact that we did not find any reinfection in this relatively small population. As mentioned earlier, there are indeed reinfections in the Belgian PWID population regardless of the very low prevalence. Furthermore, analyses could not be performed to identify potential risk factors due to the absence of reinfections. Next, HCV RNA testing by finger prick using a point-of-care molecular testing (POCT) instrument is currently not approved as a diagnostic tool in Belgium. However, the instrument has recently been validated in a population of Belgian PWID [32]. On top of that, currently, finger prick tests can only be performed by medical professionals (e.g., physicians and nurses). Instead, these finger prick tests are best performed by nonmedical professionals, such as social workers and peer supporters, as they are the ones who can reach at-risk populations in a very low-threshold manner.

## Conclusion

Reinfection after successful treatment with DAA initially appears to be very low in Belgian PWID. Nevertheless, efforts should be made to systematically screen persons with persistent risk behavior for reinfection since about one-quarter still show risk behavior after treatment. Screening for reinfection should be optimized by adjusting reimbursement for HCV RNA testing, recognizing POCT as a diagnostic tool, and legalizing the use of POCT by nonmedical professionals. In addition, a national HCV registry should be established to accurately define the burden of HCV infection and reinfection in Belgium and support the elimination of viral hepatitis C in Europe.

## Data Availability

The data supporting the conclusions of this article are included within the article.

## Abbreviations

HCV: Hepatitis C virus
Ab: Antibody
PWID: People who inject drugs
DAA: Direct-acting antivirals
OAT: Opioid agonist treatment
IDU: Injecting drug use
NSP: Needle syringe program
IQR: Interquartile range
POCT: Point-of-care molecular testing
